# Time-Sharing-Based Synchronization and Performance Evaluation of Color-Independent Visual-MIMO Communication

**DOI:** 10.3390/s18051553

**Published:** 2018-05-14

**Authors:** Tae-Ho Kwon, Jai-Eun Kim, Ki-Doo Kim

**Affiliations:** Department of Electronic Engineering, Kookmin University, Seongbuk-gu, Seoul 02707, Korea; kmjkth@kookmin.ac.kr (T.-H.K.); eun9477@kookmin.ac.kr (J.-E.K.)

**Keywords:** synchronization, visual-MIMO, symbol error rate, camera, LED array

## Abstract

In the field of communication, synchronization is always an important issue. The communication between a light-emitting diode (LED) array (LEA) and a camera is known as visual multiple-input multiple-output (MIMO), for which the data transmitter and receiver must be synchronized for seamless communication. In visual-MIMO, LEDs generally have a faster data rate than the camera. Hence, we propose an effective time-sharing-based synchronization technique with its color-independent characteristics providing the key to overcome this synchronization problem in visual-MIMO communication. We also evaluated the performance of our synchronization technique by varying the distance between the LEA and camera. A graphical analysis is also presented to compare the symbol error rate (SER) at different distances.

## 1. Introduction

Nowadays, researchers have become more interested in visual multiple-input multiple-output (MIMO) communication as a large proportion of the population currently uses smartphone cameras. Here, the visual-MIMO system indicates the visible light communication (VLC) between a light-emitting diode (LED) array (LEA) and a camera to make this kind of communication practically convenient for the user. There are also many applications of the visual-MIMO system, like V2X (vehicle-to-everything) communication. The most important point is the camera, which is always available and often used in our daily life. However, these low-price general cameras have low frame rates, whereas a transmitter LED has a high data rate. Therefore, there needs to be a way to properly synchronize them.

This work is an extended version of [1] where we proposed the synchronization technique for color-independent visual-MIMO. In this paper, the synchronization algorithm is described in detail, and at the same time, we adopt two example cases to verify our algorithm. We also compare the performance by varying the distance between transmitter and receiver. Besides, we show an application example of a real-time scenario. Several studies on visual-MIMO techniques have been conducted [1,2,3,4,5,6,7,8,9,10,11,12,13,14], and the possibility of visual-MIMO communication for real-time applications has been described in [2]. Meanwhile, the same research team has also described a multiplexing and diversity technique to adapt the visual-MIMO communication in real time [3]. In [4], LED array detection was performed using the pattern of sync-LEDs. Based on sync-LEDs, they could find out the start point of an on-off keying (OOK)-modulated data sequence and calibrate the distorted image snapshots. In [5], the paper deals with the problem of synchronization between LCD displays to smartphone camera communication. It was named after their design as “LightSync”. They used LightSync as a frame generator for the display. By adopting a linear erasure code across the original frames, they recovered the lost frames. Thus, they synchronized the data between the LCD screen and smartphone camera. In [7], they showed that transmitting simultaneously a common preamble can allow synchronization with the start bit of the VLC data packet. In their proposed scheme, they also show that the periodic nature of packet transmissions allows the receiver to synchronize with the infrastructure. In [8], they presented HiLight, a new form of real-time screen-camera communication without showing any coded images (e.g., barcodes) for off-the-shelf smart devices. In [9], they produced ColorBars by using an RGB tri-LED, and they used the color shift keying (CSK) modulation scheme. They described inter-frame data loss, which happened due to the unsynchronization between transmitter and receiver. To solve this problem, they used the error correction coding scheme. In [10], downlink VLC using a mobile phone camera is presented. An efficient rotation compensation feature was provided in the form of a special header frame called the keyframe to allow multiple users to receive the data simultaneously. In [11], they proposed a smartphone localization method based on acoustic waves and a VLC system by time synchronizing. They used the smartphone’s microphone to receive the acoustic signal and the smartphone video camera to synchronize the time. Their transmitter module also consisted of a loudspeaker and an LED. They modulated their LED signal by a square wave, which had one third the frequency of the video frame rate. This implies that the transmitter flickering rate was not larger than the frame speed, so human eyes may catch this flickering. In [14], they proposed the synchronization method without knowing the transmission period at the receiver side. They suggested a new demodulation method through minimizing the cost functions of their investigated parameters. It can be useful for OOK modulation in a VLC system.

A color-space-based modulation scheme, called generalized color modulation (GCM), for color-independent VLC systems was proposed and analyzed by us [15,16,17]. The modifier “color-independent” indicates the independence of variations in light color and intensity. By incorporating GCM into visual MIMO communication, we can obtain a better symbol error rate (SER) performance, higher data rate over a larger transmission range and, most importantly, color independency when compared with conventional LED communication. In [13], we showed the applicability of generalized color modulation (GCM)-based visual-MIMO for V2X. By using the proposed visual-MIMO scheme, while performing seamless communication, we can maintain the original color and brightness in addition to increasing the capacity by using color encoding.

In this paper, we propose an effective time-sharing synchronization method in addition to maintaining the color uniformity at a frequency where the human eye cannot detect flickering even at a camera frame rate of 30 fps for color-independent visual-MIMO communication. In other words, we propose the synchronization method best suited for our GCM-based visual-MIMO to avoid flicker and color non-uniformity. In our study, the frame rate of the video camera is 30 fps. We vary the distance between a transmitter and receiver to compare SER performance and describe our findings in the Experimental Results Section 4. To demonstrate its practical applicability, we also develop a prototype of the zipper slider type, which can be attached or detached by users.

In Section 2, we describe the related theory, while we give a description of our proposed algorithm in Section 3, and then in Section 4, we provide the experimental results. Finally, we conclude our work in Section 5.

## 2. Related Theory

### 2.1. Generalized Color Modulation

The GCM method has been proposed in order to express luminescence color and brightness differently according to the situation and to enable normal communication [15,16,17]. It is a communication method that can respond to environmental changes in real time regardless of the color and intensity of light, and its most prominent feature versus other modulation methods is color independency. Here, ‘color independency’ means that stable communication is possible regardless of variation in light color and intensity.

**Figure 1:**
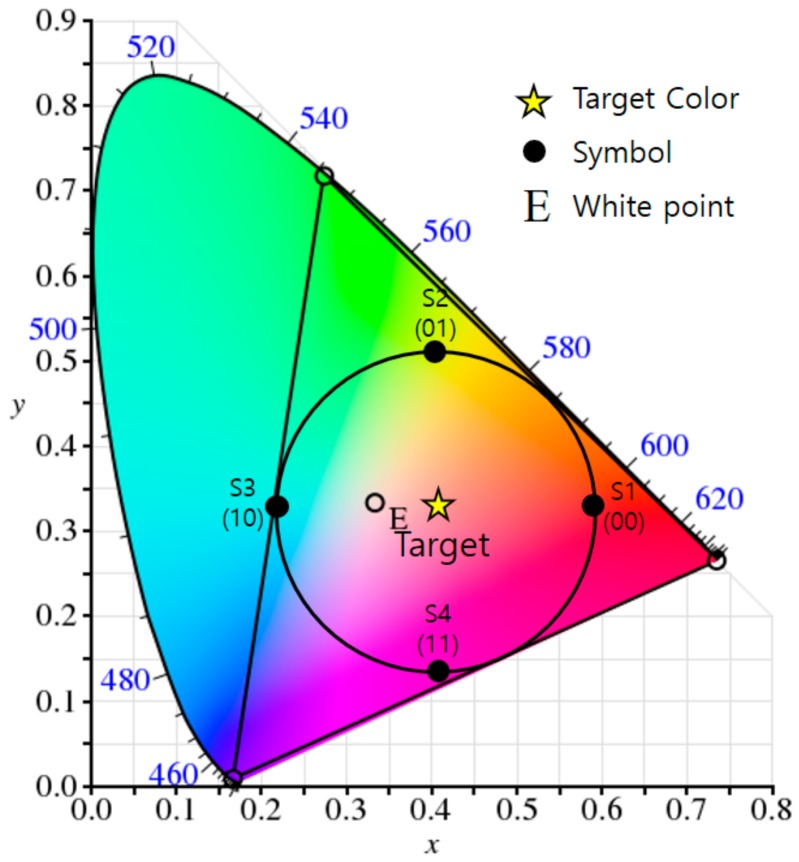
An example of the generation of a constellation diagram for a target color in the CIE1931 color space (the example uses RGB LEDs and two-bit data symbols).

GCM is able to generate any color within a gamut of them by combining some of the wavelengths or colors, and so through this, we can achieve a VLC scheme that maintains the original color and brightness while performing seamless communication. The process constructs a constellation diagram in a light color space to represent data symbols, and each constellation point in a color space represents a corresponding symbol (color). A simple example of constellation generation for a target color (i.e., the color perceived by the human eye after modulation) is illustrated in Figure 1, in which the target color is the average of all appropriate constellation points and indicates the desired color of the lighting. As can be seen, the constellation points in the color space are organized into a similar arrangement to that used in RF circular quadrature amplitude modulation (QAM).

### 2.2. Color-Independent Visual-MIMO

**Figure 2:**
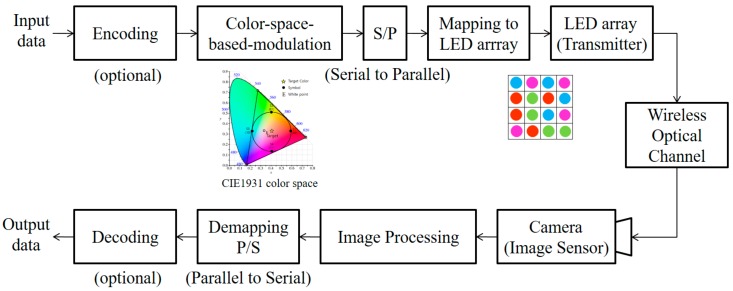
The color space-based color-independent visual-MIMO transceiving procedure using image processing.

The visual-MIMO system is a communication system between an LEA and a camera. All the LEDs in the LEA serve as transmitters, and all the pixels of the camera function as receivers [4]; thus, the optical data transmitted wirelessly by the LEA are received by the pixel array of the camera. Moreover, it is possible to increase the signal-to-noise ratio (SNR) by selecting and combining strong signals among the signals broadcast from the transmitter [3]. Recently, the possibility of a new communication method by applying the GCM method to the modulation and demodulation process of a visual-MIMO system called the color-independent visual MIMO communication system has been suggested [12,13]. Figure 2 shows a block diagram of this system based on the color space [12].

Each LED in the LEA corresponding to the transmitting end provides color information corresponding to a data symbol. As in the GCM method, the color information of the symbol designates the data in the color space-based constellation diagram. In order not to impair the role of the lighting, the color and blinking speed of each LED are specified so that the human eye cannot detect the color change of each LED occurring in the communication process. Thus, a fast data rate is needed so that the human eye cannot perceive the flashing of the LED.

An image sensor in the camera is used as the receiving end. At this time, the frame rate of the camera should be synchronized with the rate of color change of the transmitter. In order to demodulate data from the color received from the image sensor, the receiving end must have the same color space-based constellation as the transmitting end. Subsequently, the data are demodulated into the symbol of the position where the Hamming distance is at a minimum from the position of each symbol (constellation point) of the constellation diagram generated in the transmitter end.

## 3. The Proposed Algorithm

### 3.1. The Synchronization Issue

Currently, the visual-MIMO system has difficulty synchronizing, since LEDs send data at a very high rate, while the camera frame rate is low. To solve this problem in general, we should make both the data rate and frame rate at the same level, but if we increase the camera frame rate to the same level as the LED data rate, the price of a camera can become expensive. On the contrary, if we decrease the LED data rate to the level of the frame rate, flickering may be detected by the human eye, which negates the original function of the LED lighting. In this paper, we suggest an effective method to solve the problems of synchronization and flicker simultaneously by time sharing the information data with synchronization data, even though the camera frame rate is much lower than the desired data rate.

### 3.2. The Transmitter

**Figure 3:**
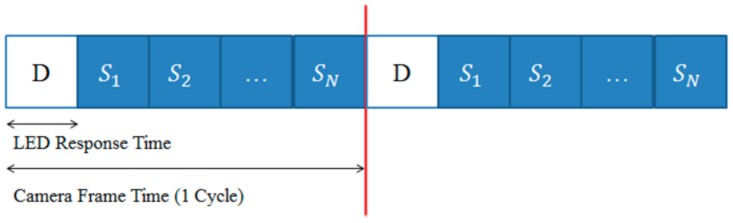
The proposed transmitted data structure.

We generate the information and synchronization data as shown in Figure 3 without violating the purpose of color-independent GCM. In the figure, the white part (D) represents the information symbol (color) data; the blue part represents the symbol data used for time synchronization; and *S*_1_–*S_N_* represent the symbols corresponding to the constellation points.

**Figure 4:**
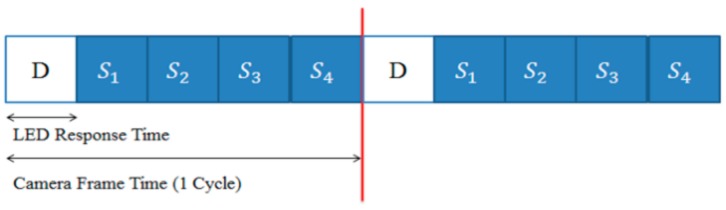
An example of the proposed transmitted data structure (the example uses two-bit data symbols).

Obviously, information data are generated randomly, and synchronization data are generated in a predetermined order. If we consider two-bit data symbols as in Figure 1, the proposed transmitted data structure can be represented as in Figure 4, in which the transmitting data rate is five-times the receiving frame rate.

**Figure 5:**
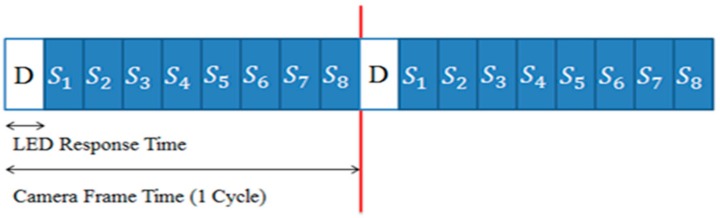
An example of the proposed transmitted data structure (the example uses three-bit data symbols).

**Figure 6:**
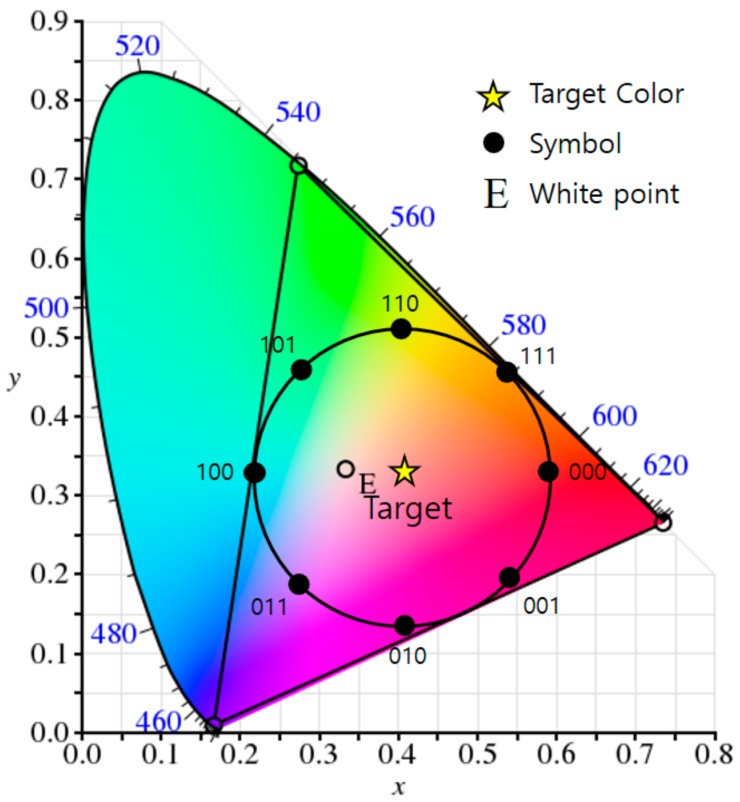
An example of generating a constellation diagram for a target color in the CIE1931 color space (the example uses RGB LEDs and three-bit data symbols).

The number of synchronization data symbols can be changed depending on the difference between the LED data rate and the camera frame rate. For example, if the speed difference between the LED and the camera is nine-times, the transmission data symbols are composed as shown in Figure 5 using eight synchronization symbols. In this case, the information symbol is constructed in the CIE1931 color space as shown in Figure 6.

Since the probability that all information data in an LEA are the same is ${\left(\frac{1}{S}\right)}^{N-1}$, where *N* is the total number of LEDs of the LEA and *S* is the total number of symbols; we can easily distinguish between the synchronization and information data if *N* is large enough.

### 3.3. The Receiver

**Figure 7:**
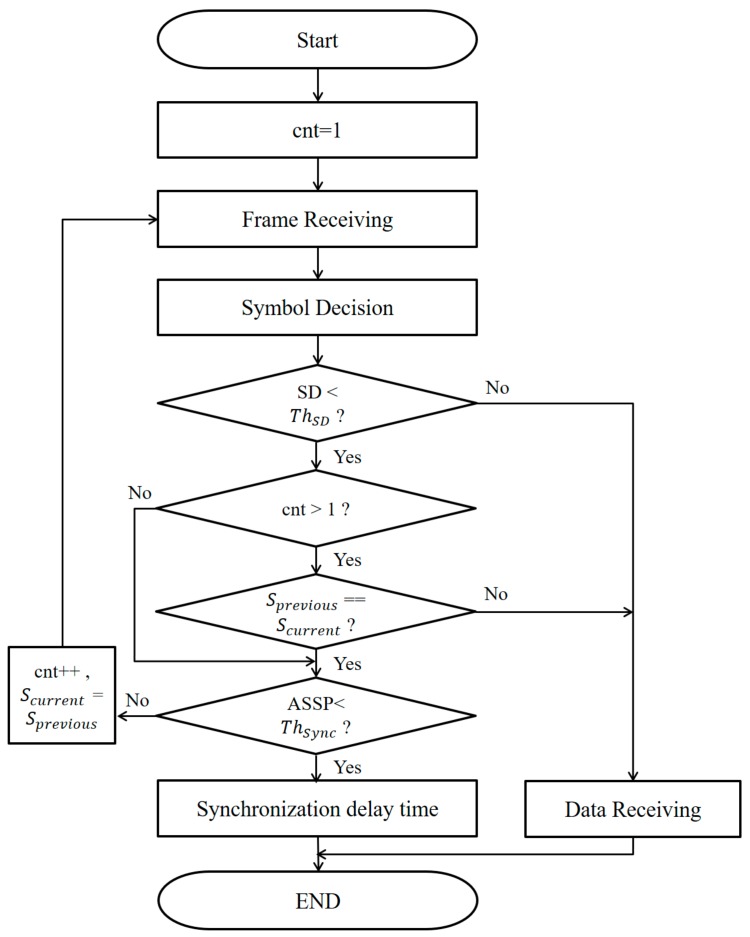
A block diagram of the proposed synchronization algorithm. ASSP, accumulated symbols same probability.

Figure 7 shows a block diagram of the proposed synchronization algorithm.

The definitions of the parameters used in Figure 7 are as follows:

- cnt: number of accumulated frames
- SD: standard deviation
- *Th_SD_*: the threshold of the standard deviation
- *S_previous_*: the symbols of the previous frame
- *S_current_*: the symbols of the current frame
- ASSP (accumulated symbols same probability): the probability that all LEDs in the accumulated frames will have the same symbol
- *Th_sync_*: the threshold probability that can be used as a decision measure of a synchronous frame

We can calculate the standard deviation (SD) of the intensity value of RGB for *N* LEDs of an LEA to determine whether the received data are for either information or synchronization as:

(1) $$SD=\frac{1}{3}\sum_{C\in \{R,G,B\}}\sqrt{\frac{\sum_{i=1}^{N}{\left({LED}_{c}(i)-{AVG}_{c}\right)}^{2}}{N}}$$

Equation (1) is obtained by averaging the SD values of the intensity of each *R*, *G* and *B* channel of all the LEDs, in which *C* represents each channel of RGB color and *N* is the total number of LEDs. Furthermore, *LED_c_*(*i*) and *AVG_c_* represent the intensity of the *i*-th LED and the average of the luminance of all LEDs, respectively.

If the SD is greater than the experimentally-determined threshold value *Th_SD_*, the received data are determined to be information data. If the SD is less than the threshold value, we first check the value of cnt. If cnt is equal to one and if ASSP is smaller than *Th_sync_*, the received data are determined to be synchronous data. However, if ASSP is larger than *Th_sync_*, cnt is incremented by one, the current symbols are stored in *S_previous_* and the next frame is received. Next, if cnt is two or more, then when *S_previous_* and *S_current_* are not the same, the received data are determined to be information data. When *S_previous_* and *S_current_* are the same and if ASSP is smaller than *Th_sync_*, the received data are determined to be synchronous data. However, if ASSP is larger than *Th_sync_*, cnt is incremented by one, the current symbol is stored in *S_previous_* and the next frame is received. The ideal ASSP value is defined as:

(2) $$ASSP=\frac{1}{S^{(cnt\times N)-1}}$$

where *S* is the number of symbols and *N* is the total number of LEDs. The threshold value of ASSP is determined by considering the channel environment. If the received data are finally determined to be synchronous data, we can determine a synchronization delay time corresponding to the received symbol as in Equation (3).

(3) $$Synchronization\;delay\;time=\frac{S+1-i}{S+1}$$

where *S* is the total number of symbols and *i* is the received symbol number. For example, when we used four symbols (*S*_1_–*S*_4_) and received *S*_1_, then the delay will be 4/5 frame. When the delay time is applied, it will be synchronized after one frame. The reason for this is that since the time for the information data in the next frame is shorter than one frame time, it is finally synchronized in the next frame after the synchronization delay time.

## 4. Experimental Results

For the experimental environment, the transmitting end consisted of an RGB LED (WS2812B, WORLDSEMI, Dongguan, China) array (4 × 4) and an ATmega328 (Atmel, AZ, USA) for the LED operation control. The receiving end comprised a Flea3 FL3-U3-13S2C camera (FLIR, Richmond, BC, Canada), and the OpenCV library was used for symbol determination and delay time application. The experimental environment is shown in Table 1.

We set the 16 LEDs to blink simultaneously at 150 Hz. The reason for this was basically that human beings can perceive the blinking phenomenon at a light emission frequency of 100 Hz, but at 150 Hz or more, they cannot consciously detect it [18].

**Figure 8:**
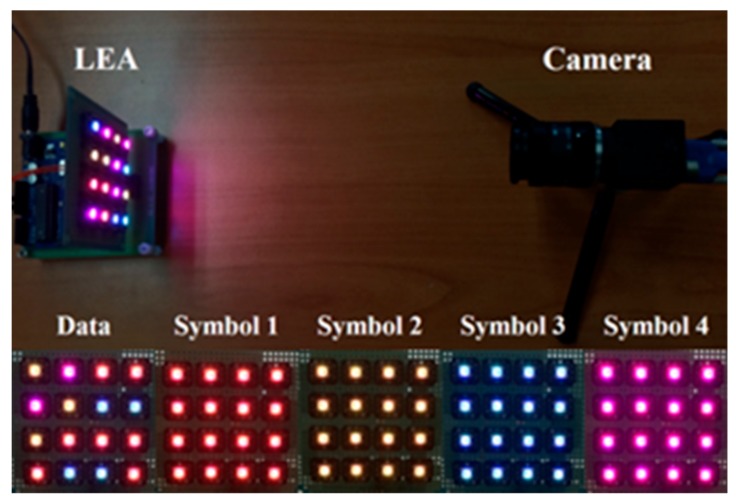
The experimental environment.

Figure 8 shows an experimental environment for the proposed method when 16 LEDs (4 × 4 LEA) were used as a transmitter. Here, two-bit data symbols are transmitted by RGB LEDs. In Figure 8, we can see that the LEA and the camera are placed on the upper left and upper right, respectively. The lower part of the figure shows an example of information data and synchronization data (four symbols, *S*_1_–*S*_4_) received through the camera.

**Figure 9:**
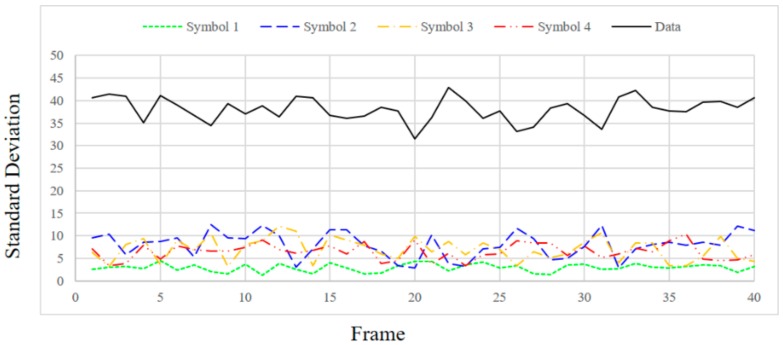
Standard deviation at a distance of 0.5 m.

**Figure 10:**
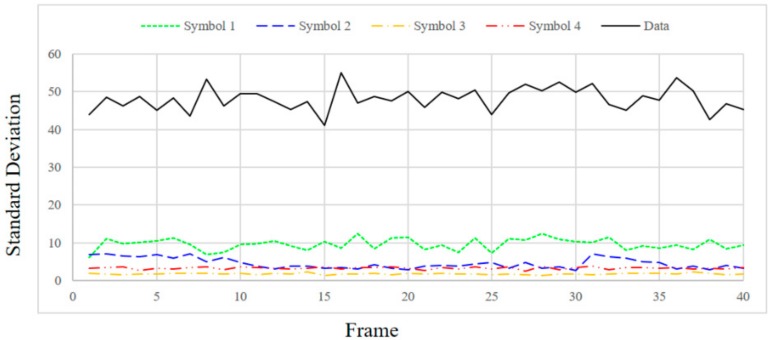
Standard deviation at a distance of 1 m.

**Figure 11:**
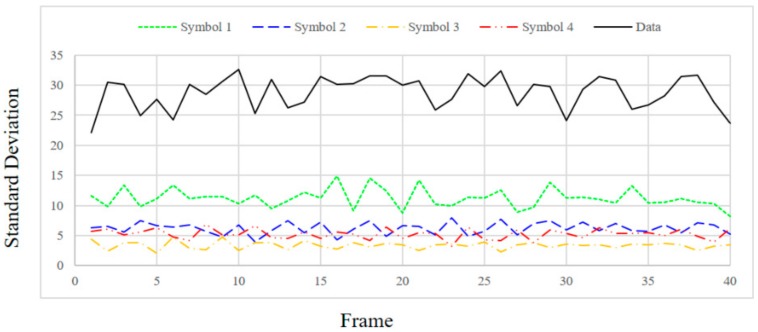
Standard deviation at a distance of 1.5 m.

**Figure 12:**
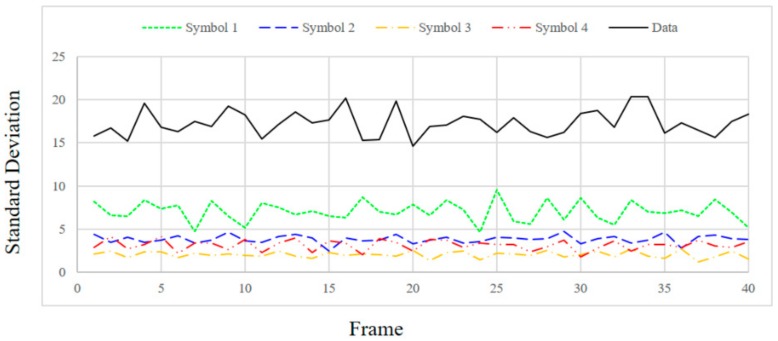
Standard deviation at a distance of 2 m.

In order to check the SD value according to the distance between the transmitting end and the receiving end, the distance was changed to 1, 1.5 and 2 m and the SD values were measured. Figure 9, Figure 10, Figure 11 and Figure 12 show the SD values when the distance between the transmitting end and the receiving end was 0.5, 1, 1.5 and 2 m, respectively.

In the case of 0.5 m, since the intensity of the emitting LED is strong, a saturated region with white is generated at the center of the LED in the received image. Therefore, since white noise is added to the color of each symbol, the standard deviation is relatively small for the information data. In the case of 1 m, since the distance between the LED and the camera is sufficiently far away, in the received image from the camera, the area saturated with white is small at the center of the LED. As a result, the color of each symbol is clearly received by the camera, so SD can be larger in the case of information data. In the case of 1.5 m or more, since the distance between the LED and the camera is too long, it affects the color of the neighboring LEDs by the light blurring of each LED in the received image from the camera. As the distance increases, the interference between adjacent LEDs increases due to color blurring. Therefore, the SD of the information data becomes smaller as the distance increases.

Experimentally, the threshold was set to 20 for stable communication at a distance of 0.5–1.5 m, and the threshold value of ASSP (*Th_sync._*) was set to 1/10,000 in consideration of the channel environment. Since the SD values of the information frames differ significantly from those of the synchronization frames, as shown in Figure 9, Figure 10 and Figure 11, we can confirm the validity of our proposed method. However, the SD value of the information data dropped to 20 or less at a distance of 2 m.

**Figure 13:**
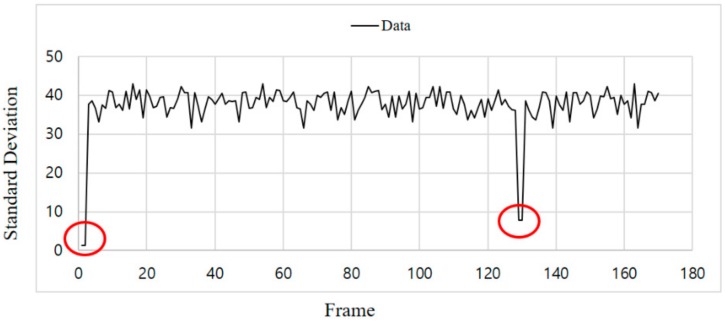
Standard deviation after applying the delay time of the synchronization scheme.

Since the proposed synchronization scheme confirms synchronization for every frame, even if synchronization is lost during communication, it can recognize this immediately and then synchronize. Figure 13 shows the standard deviation after applying the proposed synchronization scheme.

As can be seen in Figure 13, after the initial synchronization, the SD value increased to at least 30, but then decreased to less than 10 because the synchronization shifted at about the 130th frame. After this, it can be seen that synchronization took place after two frames, and the SD value increased again. Through this, it is possible not only to synchronize initially, but also to check whether every frame is synchronized during transmission and to recover even if synchronization is out of phase.

**Figure 14:**
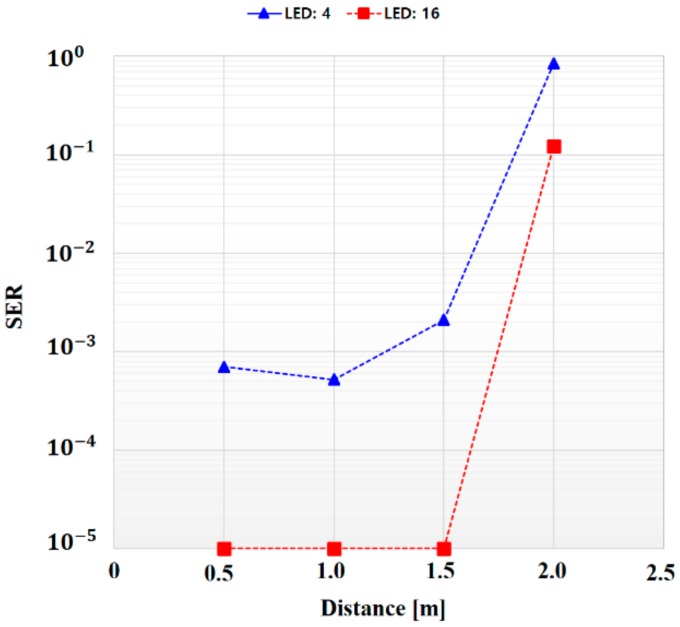
Symbol error rate according to the distance (4 × 1 LEA vs. 4 × 4 LEA).

To verify the performance of the synchronization scheme, we analyzed it in terms of symbol error rate (SER) by collecting 100,000 received data according to the distance. As can be seen in Figure 14, in order to verify the performance change of the proposed synchronization method according to the number of LEDs, SER was measured against distance when 16 LEDs (4 × 4 LEA) and 4 LEDs (4 × 1 LEA) were used for the same environment and parameters.

When 16 LEDs (4 × 4 LEA) were used, there were no errors from a 0.5–1.5 m distance, but 12,200 errors occurred at 2 m. Hence, we confirmed that it was possible to communicate effectively up to a distance of 1.5 m with the specified threshold value. In the case of using four LEDs (4 × 1 LEA), it was confirmed that 70, 52, 210 and 64,400 errors occurred at distances of 0.5, 1, 1.5 m and 2 m, respectively, among 100,000 transmitted data This indicates that a relatively large number of errors occurred compared with the case of using 16 LEDs. In the case of a 4 × 1 LEA, since the SD value of the synchronization data increased, the difference from the SD value of the information data decreased, causing synchronization errors to occur more frequently and the performance to degrade.

**Figure 15:**
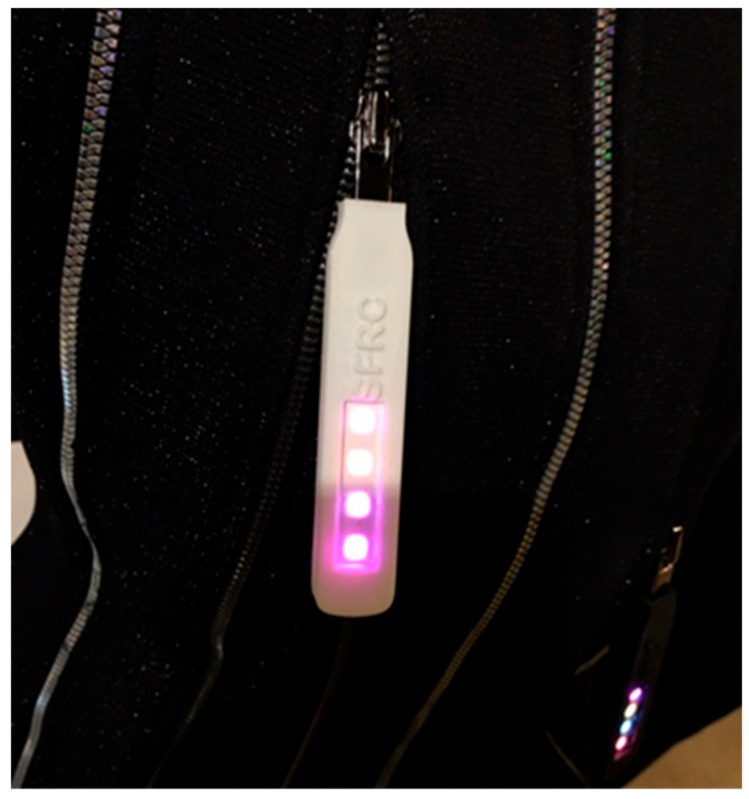
The transmitter end of the experimental zipper slider prototype.

**Figure 16:**
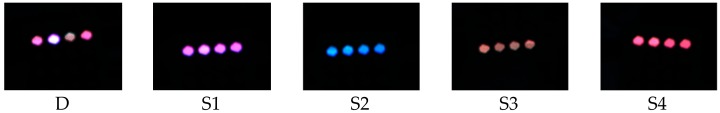
The received image of each symbol.

Figure 15 shows the implemented zipper slider prototype as a transmitter, and Figure 16 shows the example of each received symbol image. In Figure 16, D represents the information data and *S_1_−S_4_* represent the synchronization data (four symbols) received through the camera.

## 5. Conclusions

We proposed an effective time-sharing-based synchronization technique to overcome the problems of synchronization and flicker simultaneously in color-independent visual-MIMO communication, even though the camera frame rate is much lower than the desired data rate. In other words, we achieved effective synchronization in addition to maintaining color uniformity at a frequency where the human eye cannot detect flickering when the camera frame rate is much lower than the transmitted data rate. This allows LEAs to maintain high speed, which not only impairs the functionality of the desired light source, but also shows the possibility of communicating with a relatively low-speed commercial camera. We generated the information and synchronization data without violating the purpose of color-independent GCM. We used the standard deviation of the intensity value of RGB for *N* LEDs of an LEA to determine whether the received data are for either information or synchronization. Through the experimental results, we confirmed the validity of our proposed method. To evaluate the performance of our synchronization technique, we varied the distance between the LEA and camera, and a graphical analysis was presented to compare the SER for different distances, as well as different numbers of LEDs. To demonstrate the practical applicability, we also developed a prototype of the zipper slider types, which can be attached or detached by users.

## Figures and Tables

**Table 1:** Experimental environment.

	Name (Information)
LED array	RGB LED WS2812B (4 × 4, 150 Hz)
Embedded	Arduino Uno (Atmega328)
Camera	Flea3 FL3-U3-13S2C (30FPS)
System	Win7 (64 bit)
Software	Visual studio 2013 (OpenCV)
